# Left atrial epicardial adipose tissue is associated with low voltage zones in the left atrium in patients with non-valvular atrial fibrillation

**DOI:** 10.3389/fcvm.2022.924646

**Published:** 2022-07-14

**Authors:** Yameng Shao, Lei Chen, Wensu Chen, Chuanyi Sang, Changjiang Xu, Chaoqun Zhang

**Affiliations:** ^1^Department of Cardiology, The Affiliated Hospital of Xuzhou Medical University, Xuzhou, China; ^2^Department of Cardiology, The Affiliated Huai’an No. 1 People’s Hospital of Nanjing Medical University, Huai’an, China

**Keywords:** atrial fibrillation, epicardial adipose tissue, fibrosis, low voltage zones, radio frequency catheter ablation

## Abstract

**Objective:**

Epicardial adipose tissue (EAT) is related to atrial fibrillation (AF), but the specific mechanism is still unclear. Left atrial (LA) low voltage zones (LVZ) can well reflect atrial fibrosis. This study investigated the relationship between EAT and LVZ in non-valvular AF (NVAF) patients.

**Methods:**

This observational study including patients with NVAF (*n* = 214) undergoing radiofrequency ablation (RFCA) for the first time in our hospital and 62 matched controls. The EAT volume and attenuation were measured by contrast-enhanced computed tomography. A three-dimensional mapping system was used to map the left atrial endocardium and evaluate LA-LVZ. Patients were divided into LVZ and non-LVZ groups according to the presence or absence of LVZ.

**Results:**

Patients with AF showed higher LA-EAT volume and lower attenuation value than controls (29.7 ± 11.2 cm^3^ vs. 20.9 ± 8.6 cm^3^, *P* = 0.021; −91.2 ± 5.6 HU vs. −88.7 ± 5.9 HU, *P* < 0.001). Compared with the group without LVZ, there were significant differences in age [65 (59–71) vs. 60 (52–69), *P* = 0.006], LAVI [75.1 ± 20.7 ml/m^2^ vs. 67.2 ± 20.9 ml/m^2^, *P* = 0.018], LA-EAT volume (34.8 ± 11.5 cm^3^ vs. 28.1 ± 10.6 cm^3^, *P* < 0.001) and LA-EAT attenuation (−93.9 ± 5.3 HU vs. −90.4 ± 5.5 HU, *P* < 0.001). Multivariate regression analysis showed that age (OR = 1.040; 95%*CI*: 1.001–1.078, *P* = 0.042), LAVI (OR = 1.019; 95%*CI*: 1.002–1.037, *P* = 0.032), LA-EAT volume (OR = 1.193; 95%*CI*: 1.015–1.402, *P* = 0.034) and attenuation value (OR = 0.801; 95%*CI*: 0.701–0.916 *P* = 0.001) were independent predictors of LVZ. After LA-EAT attenuation was incorporated into the clinical model, the comprehensive discrimination and net reclassification tended to improve (IDI and NRI > 0, *P* < 0.05).

**Conclusion:**

LA-EAT volume and attenuation values can independently predict the presence of LVZ, and LA-EAT attenuation has a better predictive value than LA-EAT volume.

## Introduction

Atrial fibrillation (AF) is the most common arrhythmia with clinical significance. Complications such as heart failure, thromboembolism, and the risk of death are significantly increased in patients with AF (1). In recent years, obesity has been considered a significant risk factor for AF (2, 3). Although subcutaneous adipose and visceral adipose are both adipose tissue, the two structures are different. Compared with subcutaneous adipose tissue, visceral adipose tissue plays an essential role in cardiovascular disease by secreting higher levels of pro-inflammatory adipokines (4–6). Epicardial adipose tissue (EAT) is a particular form of visceral adipose tissue. Because there is no myofascial separation between EAT and myocardium, EAT shares the same coronary circulation and has local and systemic effects (7, 8). More and more evidence showed that EAT is related to the occurrence and recurrence of AF (9, 10), but the specific mechanism is unclear. Basic studies have shown that EAT can induce atrial fibrosis by secreting various inflammatory or fibrogenic mediators (11, 12). It is well known that atrial structural remodeling characterized by atrial fibrosis is the core of the maintenance mechanism of AF (13). The main ways to evaluate atrial myocardial fibrosis are endocardial biopsy, intracardiac voltage mapping, and Cardiac Magnetic Resonance (CMR) imaging (14). Intracardiac voltage mapping is widely used because of its high accuracy and safety (15, 16). In this study, CT was used to determine the EAT volume and attenuation in patients with non-valvular AF, and left atrial voltage was determined by left atrial voltage mapping under the guidance of the intraoperative CARTO3 three-dimensional mapping system to explore the relationship between EAT and Left atrial low voltage zones (LA-LVZ) in patients with non-valvular AF (NVAF).

## Materials and methods

### Study population

**FIGURE 1:**
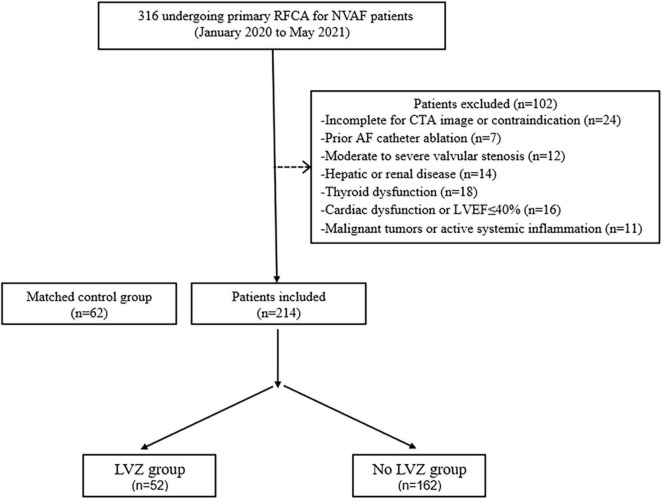
Flow chart detailing the inclusion and exclusion criteria.

This is a single-center retrospective clinical observation study. We systematically reviewed 316 patients with NVAF who were admitted to the affiliated Hospital of Xuzhou Medical University for the first time from January 2020 to May 2021. The definition of AF is consistent with current guidelines (17). All patients received a CTA examination of the left atrium and pulmonary vein before the operation. Exclusion criteria: (1) Incomplete for CTA image or contraindication; (2) Sinus rhythm cannot be restored after radiofrequency catheter ablation; (3) Previous history of radiofrequency catheter ablation; (4) History of rheumatic valvular disease, moderate to severe valvular stenosis or insufficiency, congenital heart disease; (5) Cardiac dysfunction; (6) Severe hepatic and renal insufficiency, thyroid dysfunction, respiratory disease, and history of the malignant tumor. A total of 214 patients with NVAF were finally included (Figure 1). This group was compared to 62 matched (age, sex, BMI) controls who underwent multidetector computed tomography for screening of CAD and had no history of AF (Figure 1).

The ethics committee of the Affiliated Hospital of Xuzhou Medical University has reviewed the research scheme, and all patients have signed informed consent forms.

### Cardiac computed tomography imaging

Spiral CT (SOMATOM Definition, SIEMENS Germany) to obtain CT imaging data. 60–80 ml iohexol was injected into the elbow vein at the flow rate of 5 ml/s, and then saline of 50 ml was injected at the speed of 5 ml/s. The contrast agent tracking technique triggered the enhanced scan—trigger plane: ascending aorta root level; trigger threshold: 90∼100 Hu. The scanning time was 5∼12 s after 6 s delay, and the scanning range was from 1 cm under the trachea Carina to 1.5 cm of the inferior margin of the heart. Scanning parameters: tube current 280∼350 mA, tube voltage 120 kV.

### Image post-processing technology and observation method of measurement

**FIGURE 2:**
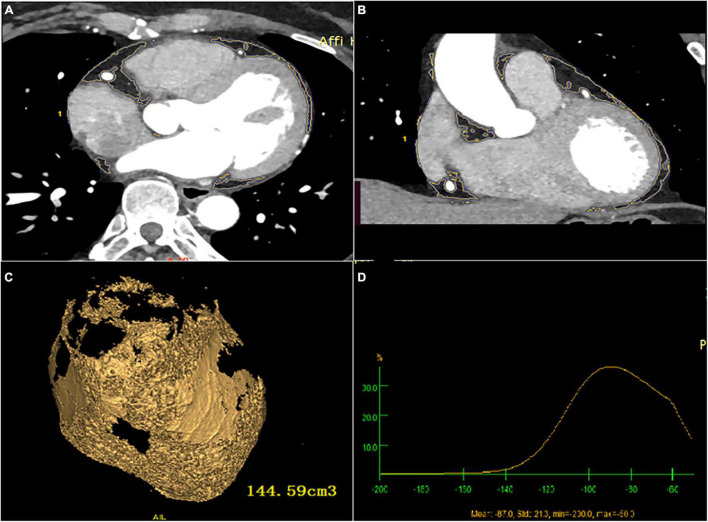
Total EAT volume and attenuation calculated by post-processing software **(A)** cardiac axial map; **(B)** cardiac coronal map; **(C)** yellow area represents total EAT, and the volume is calculated by Volume software; **(D)** total EAT attenuation.

**FIGURE 3:**
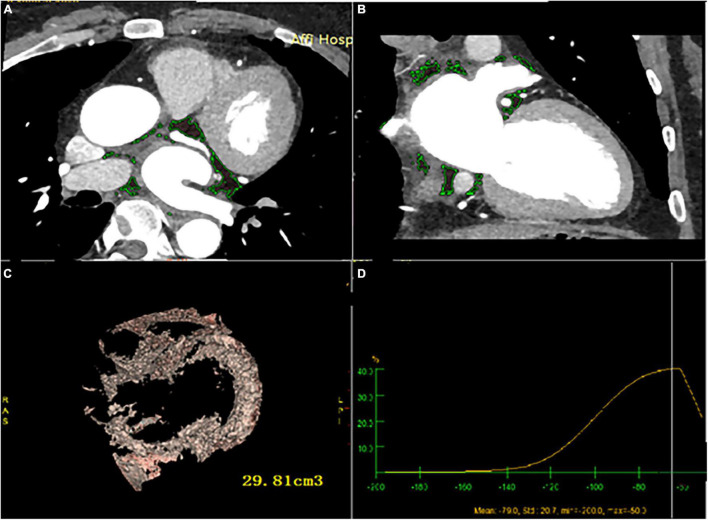
LA-EAT volume and attenuation calculated by post-processing software **(A)** cardiac axial map; **(B)** cardiac coronal map; **(C)** yellow area represents LA- EAT, and the volume is calculated by Volume software; (D) LA- EAT attenuation.

All images were reconstructed by retrospective ECG-gated reconstruction, slice thickness 0.5 mm, overlapping 0.3 mm, visual reconstruction field was adjusted according to the patient’s physique and heart size, and the heart volume data of 75% of the Rmurr gap was reconstructed. All data are sent to (Advantage workstation GE 3.2 United States workstation) for image post-processing. The axial images from the bifurcation of the pulmonary artery to the apical were semi-automatically reconstructed from the adjacent 0.625 mm slices. The volume was measured by combining the slice method with the threshold method. Firstly, the cardiac parietal and visceral layers were found, the epicardial boundary was outlined layer by layer, the CT value of −50 HU ∼ −200 HU was set as adipose tissue, and the total EAT, and radiation attenuation were calculated automatically (Figure 2). After that, the pericardial adipose in the left ventricle in front of the mitral annulus, the epicardial adipose in the right atrium in front of the right superior pulmonary vein, and the epicardial adipose below the plane of the coronary sinus were manually removed, and the remaining epicardial adipose was LA-EAT. The LA-EAT volume and radiation attenuation were calculated (Figure 3). For the purposes of this analysis, the LA was segmented as posterior LA, anterior LA, septal LA, inferior LA, lateral LA, and LA roof, as previously described (18). Left atrial volume (LAV) and left atrial volume index (LAVI) were calculated by volume reconstruction of the left atrium, which was defined as left atrial volume divided by body surface area. The volume and attenuation of LA EAT were measured offline by two clinicians who did not know the clinical data of the patients.

### Detection of the low voltage zone

All patients underwent circumferential pulmonary vein isolation (CPVI) first and then left atrial voltage was mapped in sinus rhythm. The voltage points are collected using the CONFIDENCE Module (Biosense Webster, Inc., Diamond Bar, CA, United States) and mapped continuously using the following settings: 5% CL filtering, 3 ms LAT-stability, 3 mm position stability, and 1 mm density. For each marked site, steady contact is required between the local atrial tissue and each pair of electrodes of the Lasso catheter. Then, use the ablation catheter (ThermoCool SF, Biosense Webster, Inc., Diamond Bar, CA, United States) to confirm the LVZ. Steady contact is also needed between the local atrial tissue and the tip of parallel alignment of the ablation catheter. LVZ was defined as sites of ≥ 3 adjacent low-voltage points < 0.5 mV (15).

### Statistical analysis

If the measurement data are normally distributed, they are expressed as the mean ± SD. If they do not conform to a normal distribution, they are described by the median (quartile spacing), that is, M (Q25 Q75), and the counting data are described by the number (percentage). The counting data were compared by *t*-tests or rank-sum tests (non-normal distribution), and the classified variables were compared by chi-square tests or Fisher’s exact test. Pearson or Spearman correlation tests evaluated the relationship between LA-EAT and various parameters. A ROC curve analysis was carried out to define a cut-off value. LA-EAT volume and attenuation were examined per 5 cm^3^ and 2 HU increase respectively. The association between clinical, laboratory, echocardiographic and LA-EAT variables, and LVZ was assessed in univariable regression analyses. Variables demonstrating a significant association with the LVZ (*P* < 0.05) were included in a multivariable model performed using forward stepwise logistic regression analysis.

Additionally, 60 random studies were assessed for interobserver and intraobserver agreement. Intraobserver and interobserver reliability measurements and agreement between reference and proposed methods were assessed with the intraclass correlation coefficient (ICC). Additionally, Reproducibility was appraised with Bland–Altman analysis and reported as bias and coefficient of repeatability. The mean difference was presented as the bias, and 95% limits of agreement around the bias were expressed as the mean difference of 1.96 ± SDs.

LA-EAT volume or attenuation was added to the starting clinical model. To assess the incremental predictive value of LA-EAT volume or attenuation to clinical models, all variables in the model were included as continuous variables with additional discrimination of risk factors evaluated using the c-index and the integrated discrimination improvement (IDI). Continuous net reclassification index (NRI) was calculated, with IDI and NRI > 0 indicating positive improvement, and each model was calculated and compared. *P* < 0.05 was considered statistically significant. All statistical analyses were carried out using SPSS version 24.0 software (SPSS, Chicago, IL, United States).

## Results

### Characteristics of the study population

62 patients fulfilled the inclusion criteria and were enrolled in the study (Figure 1). Gastrointestinal disease incidence is higher in the AF group. Patients with AF had a significantly higher EAT volume and lower attenuation value than the control group in Table 1. According to the intraluminal voltage mapping results under the guidance of the intraoperative CARTO3 three-dimensional mapping system, the patients of AF were divided into the LVZ group and the non-LVZ group. The basic clinical data of patients in the two groups are shown in Table 2. Patients with LVZ were older (*P* = 0.006). There was more persistent AF (*P* = 0.021) and a significantly higher LAVI in the LVZ group compared with the no LVZ group (*P* = 0.018). The remaining variables were not statistically different (*P* > 0.05).

**TABLE 1:** Demographics and baseline characteristics: controls and patients with atrial fibrillation.

	Control group	AF group	*P*-value
	(*n* = 62)	(*n* = 214)	
Age (years)	61 (56–67)	62 (54–69)	0.774
Male, n (%)	38 (61.3)	143 (66.8)	0.450
BMI (kg/m^2^)	25.4 ± 2.5	25.1 ± 3.1	0.436
Hypertension, n (%)	27 (43.5)	89 (41.6)	0.884
Coronary disease, n (%)	10 (16.1)	33 (15.4)	0.845
Diabetes mellitus, n (%)	36 (19.4)	36 (16.8)	0.704
Gastrointestinal disorders, n (%)	4 (6.5)	36 (16.8)	0.042
LVEF (%)	59.5 ± 4.6	58.9 ± 5.4	0.438
E/e’	10.1 ± 4.3	10.9 ± 4.1	0.139
LAVI (mL/m^2^)	54.9 ± 13.8	69.1 ± 21.1	< 0.001
LVMI (g/m^2^)	89.2 ± 15.4	93.1 ± 17.6	0.119
CRP (mg/L)	1.1 ± 1.5	1.5 ± 2.6	0.264
eGFR (mL/min/1.73 m^2^)	102.57 ± 14.58	100.40 ± 16.51	0.358
Total-EAT volume (cm^3^)	124.15 ± 39.4	139.8 ± 45.9	0.016
Total-EAT attenuation (HU)	−91.2 ± 5.7	−94.5 ± 6.6	< 0.001
LA-EAT volume (cm^3^)	20.9 ± 8.6	29.7 ± 11.2	< 0.001
LA-EAT attenuation (HU)	−88.7 ± 5.9	−91.2 ± 5.6	< 0.001

Gastrointestinal disorders, gastroesophageal reflux and/or inflammatory bowel diseases; BMI, body mass index; LVEF, left ventricular ejection fraction; E, diastolic early transmitral flow velocity; e’, diastolic early mitral annular velocity; LAVI, left atrial volume index; LVMI, left ventricular mass index; LA-EAT, left atrial epicardial adipose tissue.

**TABLE 2:** Comparison of clinical and anatomic variables in patients with and without LVZ in atrial fibrillation.

	AF		*P*-value
	**LVZ (*n* = 52)**	**No LVZ (*n* = 162)**	
Age (years)	65 (59–71)	60 (52–69)	0.006
Male, n (%)	31 (59.6)	112 (69.1)	0.205
BMI (kg/m^2^)	25.3 ± 3.2	25.4 ± 2.9	0.604
CHA_2_DS_2_-VASc score	2 (1–3)	1 (1–2)	0.455
Non-paroxysmal AF, n (%)	29 (55.9)	61 (37.3)	0.021
AF duration (months)	36 (10.7–69)	24 (8.7–36)	0.091
Hypertension, n (%)	21 (40.4)	68 (42.0)	0.840
Coronary disease, n (%)	6 (11.5)	27 (16.7)	0.373
Diabetes mellitus, n (%)	13 (25.0)	23 (14.2)	0.070
Gastrointestinal disorders, n (%)	7 (13.5)	29 (17.9)	0.456
Beta-blocker, n (%)	18 (34.6)	77 (47.5)	0.103
Propanone, n (%)	14 (26.9)	42 (25.9)	0.887
Amiodarone, n (%)	23 (44.2)	66 (40.7)	0.657
LVEF (%)	59.1 ± 5.3	58.4 ± 5.8	0.411
E/e’	11.8 ± 4.8	10.7 ± 3.9	0.089
LAVI (mL/m^2^)	75.1 ± 20.7	67.2 ± 20.9	0.018
LVMI (g/m^2^)	95.4 ± 19.2	92.4 ± 17.1	0.381
CRP (mg/L)	1.9 ± 3.4	1.4 ± 2.3	0.210
eGFR (mL/min/1.73 m^2^)	103.85 ± 13.95	99.58 ± 17.02	0.220
Total-EAT volume (cm^3^)	146.4 ± 47.1	137.4 ± 45.4	0.229
Total-EAT attenuation (HU)	−95.6 ± 6.4	−94.1 ± 6.7	0.159
LA-EAT volume (cm^3^)	34.8 ± 11.5	28.1 ± 10.6	< 0.001
LA-EAT attenuation (HU)	−93.9 ± 5.3	−90.4 ± 5.5	< 0.001

Gastrointestinal disorders, gastroesophageal reflux and/or inflammatory bowel diseases; BMI, body mass index; LVEF, left ventricular ejection fraction; E, diastolic early transmitral flow velocity; e’, diastolic early mitral annular velocity; LAVI, left atrial volume index; LVMI, left ventricular mass index; LA-EAT, left atrial epicardial adipose tissue.

### Epicardial adipose tissue

There were no statistically significant differences in total eat volume (146.4 ± 47.1 vs. 137.4 ± 45.4 cm^3^, *P* = 0.229) and Total EAT attenuation value (−95.6 ± 6.4 Hu vs. −94.1 ± 6.7 Hu, *P* = 0.159) between the two groups. LA-EAT volume was larger in the LVZ group (34.8 ± 11.5 vs. 28.1 ± 10.6 cm^3^, *P* < 0.001), and attenuation was reduced (−93.9 ± 5.3 Hu vs. −90.4 ± 5.5 Hu, *P* < 0.001) compared with the no LVZ group (Table 2).

### Measurements reproducibility and agreement

**FIGURE 4:**
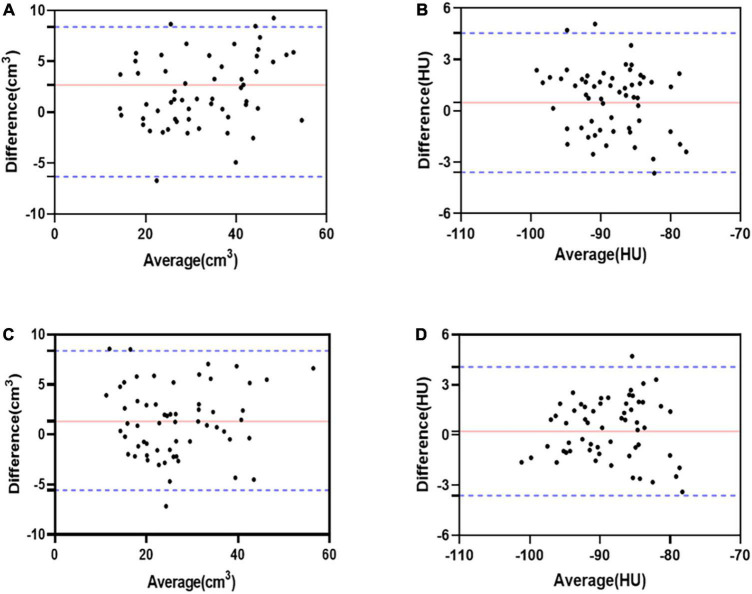
Bland-Altman bias and limits of agreement for LA-EAT volume and attenuation between interobserver. LA-EAT volume, with a bias of 2.68 ± 4.61 cm^3^ and the 95% limits of agreement was –6.34–11.7 cm^3^ **(A)**; LA-EAT attenuation, with a bias of 0.47 ± 2.07 HU and the 95% limits of agreement was –3.59–4.55 HU **(B)**. Bland-Altman bias and limits of agreement for LA-EAT volume and attenuation between intraobserver. LA-EAT volume, with a bias of 1.32 ± 3.51 cm^3^ and the 95% limits of agreement was –5.57–8.21 cm^3^ **(C)**; LA-EAT attenuation, with a bias of 0.23 ± 1.96 HU and the 95% limits of agreement was –3.61–4.07 HU **(D)**.

The ICC values for interobserver and intraobserver measurements of LA-EAT volume and attenuation were excellent correlations (Table 3). The Bland-Altman analysis showed a good agreement between interobserver and intraobserver measurement variations (Figure 4).

**TABLE 3:** Interobserver and intraobserver variability measurement.

	Interclass correlation coefficient	
	**Interobserver**	**Intraobserver**
**Two Readers (60 patients)**		
LA-EAT volume (cm^3^)	0.92 (95% CI, 0.86–0.96)	0.96 (95% CI, 0.93–0.97)
LA-EAT attenuation (HU)	0.93 (95% CI, 0.89–0.95)	0.95 (95% CI, 0.91–0.96)

LA-EAT, left atrial epicardial adipose tissue.

### The distribution of left atrial-epicardial adipose tissue

In patients with NVAF, EAT was mainly distributed in the pulmonary veins, left atrial septum, left lateral atrial wall, anterior wall, and roof. The pulmonary vein was involved in 86.9% of cases, the anterior LA in 81.3%, the septum in 77.1%, the inferior wall in 82.2%, the posterior LA in 52.3%, the lateral LA in 87.4%, and the Mitral isthmus in 95.3%.

### Clinical correlates of epicardial adipose tissue volume and attenuation

The patients with non-paroxysmal AF had larger LA-EAT volume (32.8 ± 11.1 cm^3^ vs. 27.1 ± 10.2 cm^3^, *P* < 0.001) and lower LA-EAT attenuation (−91.9 ± 5.7 HU vs. −89.8 ± 5.3 HU, *P* < 0.006). The attenuation value of LA-EAT in females was lower than that in males (−91.9 ± 5.3 HU vs. −90.1 ± 5.7 HU, *P* = 0.020), but there was no difference in LA-EAT volume. Linear correlation analysis showed that age (*r* = 0.177), BMI (*r* = 0.268) and LAVI (*r* = 0.247) correlated with LA-EAT volume, but had no significant correlation with LA-EAT attenuation (Table 4).

**TABLE 4:** Correlation between LA-EAT volume and attenuation and clinical variables.

	LA-EAT volume		LA-EAT attenuation	
	β	***P*-value**	β	***P*-value**
Age (years)	0.177	0.042	−0.151	0.121
Female, n (%)	0.296	0.432	−0.216	0.025
BMI (kg/m^2^)	0.268	< 0.001	−0.031	0.625
Diabetes mellitus, n (%)	0.127	0.060	−0.076	0.271
AF pattern	0.285	< 0.001	−0.154	0.031
CHA_2_DS_2_-VASc score	0.220	0.247	0.101	0.147
LVEF%	−0.182	0.124	−0.034	0.661
LAVI (ml/m^2^)	0.253	0.034	−0.057	0.403
CRP (mg/L)	0.076	0.270	0.101	0.141

BMI, body mass index; LVEF, left ventricular ejection fraction; LAVI, left atrial volume index; LVMI, left ventricular mass index; LA-EAT, left atrial epicardial adipose tissue; AF, atrial fibrillation; hs-CRP, high sensitivity c-reactive protein.

### The prevalence and distribution of low voltage zones

**FIGURE 5:**
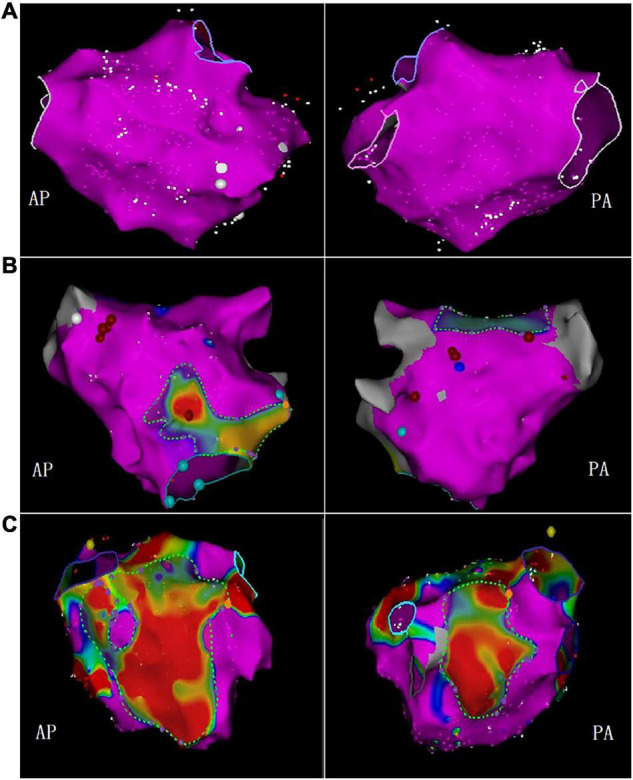
The bipolar voltage was used to measure the LVZ. The purple area represented the standard atrial matrix, and the color area represented the LVZ area. **(A)** No LVZ; **(B)** mild LVZ; **(C)** severe LVZ (AP, anterior-posterior position; PA, posterior-anterior position).

**FIGURE 6:**
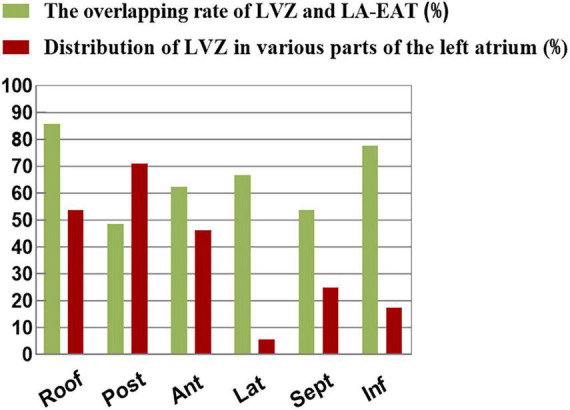
The distribution of LVZ in each segment of the left atrium and the consistency of overlap with LA-EAT.

Left atrial voltage mapping was completed in all patients under sinus rhythm after CPVI, with an average of 674 ± 82 marked points in each case. LVZ was detected in 52 patients (24.3%). In the LVZ group, the posterior wall was 71.2%, the roof was 53.8%, the anterior wall was 46.2%, the septum was 25.0%, the inferior was 17.3%, and the lateral was 5.7%. Figure 5 shows the voltage mapping results of a typical patient. Figure 6 shows the distribution of LVZ in each segment of the left atrium and the consistency of overlap with LA-EAT.

### Predictors of left atrial-low voltage zones

Univariate analysis showed that the patients in the LVZ group were older and had more non-paroxysmal AF (*P* < 0.01). In addition, LAVI and LA-EAT in the LVZ group were higher than those in the non-LVZ group. Multivariate analysis showed that advanced age (OR = 1.040; 95%*CI*: 1.002–1.080, *P* = 0.041), LAVI (OR = 1.019; 95%CI: 1.002–1.037, *P* = 0.032), LA-EAT volume (OR = 1.193; 95%*CI*: 1.015–1.402, *P* = 0.034) and attenuation (OR = 0.801; 95%*CI*: 0.701–0.916, *P* = 0.001) were independent predictors of LVZ (Table 5). From Youden index analysis, the cut-off value for LA-EAT volume was 30.0 cm^3^, and for EAT attenuation was −90.5 HU. In multivariable analysis, increased EAT volume and decreased EAT attenuation were independently associated with LVZ (Table 5).

**TABLE 5:** Association of patient characteristics with LVZ: univariate and multivariate regression analysis.

Variable	Univariate		Multivariate			
			Model 1		Model 2	
	HR (95% CI)	*P*-value	HR (95% CI)	*P*-value	HR (95% CI)	*P*-value
Age (years)	1.052 (1.016–1.088)	0.004	1.040 (1.001–1.079)	0.042	1.042 (1.004–1.081)	0.029
Female, n (%)	1.517 (0.795–2.897)	0.206				
Non-paroxysmal AF, n (%)	2.088 (1.109–3.031)	0.023				
AF duration (months)	1.005 (0.998–1.012)	0.108				
Hypertension, n (%)	1.068 (0.565–2.017)	0.840				
Diabetes, n (%)	2.014 (0.935–4.339)	0.074				
Gastrointestinal disorders, n (%)	1.402 (0.575–3.420)	0.458				
Hs-CRP (mg/L)	1.069 (0.959–1.192)	0.228				
eGFR (mL/min/1.73 m^2^)	1.012 (0.990–1.035)	0.283				
LVEF (%)	0.976 (0.922–1.034)	0.409				
E/e’	1.067 (0.990–1.149)	0.090				
LAVI (mL/m^2^)	1.018 (1.003–1.033)	0.020	1.019 (1.002–1.037)	0.032	1.017 (1.001–1.035)	0.044
LA-EAT volume, per 5 cm^3^	1.323 (1.138–1.538)	< 0.001	1.193 (1.015–1.402)	0.034		
LA-EAT attenuation, per 2 HU	0.784 (0.690–0.890)	< 0.001	0.801 (0.701–0.916)	0.001		
LA-EAT volume > 30.0 cm^3^	2.747 (1.432–5.271)	0.002			2.169 (1.086–4.330)	0.028
LA-EAT attenuation < −90.5 HU	3.635 (1.662–7.953)	0.001			3.504 (1.538–7.985)	0.003

LVEF, left ventricular ejection fraction; E, diastolic early transmitral flow velocity; e’, diastolic early mitral annular velocity; LAVI, left atrial volume index; hs-CRP, high sensitivity c-reactive protein; eGFR, estimated glomerular filtration rate; LA-EAT, left atrial epicardial adipose tissue; AF, atrial fibrillation.

### Discrimination and reclassification accuracy of the predictors of low voltage zones

The constructed clinical model includes two risk factors, age and LAVI, and the volume and attenuation of LA-EAT are added into the constructed clinical model, respectively. When LA-EAT volume (AUC = 0.725) or LA-EAT attenuation (AUC = 0.747) were included in the model, the predictive power of left atrial LVZ was significantly improved. Although IDI showed an increase of 0.046 (*P* = 0.013) after LA-EAT volume was included, the NRI was not (0.156, *P* = 0.162). When LA-EAT attenuation was incorporated into the model, both IDI and NRI were improved (IDI 0.072, *P* = 0.043; NRI 0.183, *P* < 0.001) (Table 6).

**TABLE 6:** Discrimination accuracy and reclassification of risk markers of LA-LVZ.

	C-index (95% *CI*)	IDI (95% *CI*)	*P*	NRI (95% *CI*)	*P*
Continuous markers					
Clinical models	0.667 (0.589–0.745)	–	–	–	–
+ LA-EAT volume	0.725 (0.654–0.796)	0.046 (0.010–0.083)	0.013	0.156 (−0.063–0.375)	0.162
+ LA-EAT attenuation	0.747 (0.675–0.819)	0.072 (0.033–0.109)	< 0.001	0.183 (−0.004–0.371)	0.043

CI, confidence interval; LA-EAT left atrial epicardial adipose tissue; IDI, integrated discrimination improvement; NRI, continuous net reclassification index. The established model included all the clinical risk markers for LA-LVZ which remained significant predictors of LVZ in the Cox multivariate hazards model (age, LAVI). The biomarkers LA-EAT volume and attenuation were added to the model separately and together. The analyses were performed both with all markers as continuous.

## Discussion

The main findings of this study are as follows: (1) Patients with AF showed higher volume of EAT and lower attenuation value. (2) Compared with the group without LVZ, the volume of LA-EAT in the LVZ group was more extensive, and the attenuation was lower. (3) After adjusting the clinical risk factors of left atrial LVZ, advanced age, increased LA-EAT volume and decreased radiation attenuation were independent risk factors for left atrial fibrosis. (4) LA-EAT attenuation is more valuable than LA-EAT volume prediction.

Many studies have discussed the distribution of LVZ in patients with AF, and different studies have reported different results. Hori et al. (19) used the combination of electro anatomic mapping and CT to find that the areas involved in LVZ are often concentrated in the anterior wall, posterior wall, and the area around the orifice of the pulmonary vein of LA. In this study, LVZ is mainly located in the posterior wall, in addition, the posterior wall of a single diseased segment is still the most common remodeling site, but the lateral wall and septum are not found, which is consistent with the previous findings of Radoslaw et al. (20). Possible explanations are as follow. (1) The baseline characteristics of patients in the study are not consistent. In our study, more patients have paroxysmal AF (57.9%). Theoretically, with the prolongation of the course of the disease, patients with persistent AF will have secondary structural remodeling, leading to the expansion of low-voltage areas. We found that the most common site of left atrial fibrosis in patients with paroxysmal AF is the posterior wall, suggesting that posterior wall fibrosis may be a cause rather than a result of AF. Intervention on the posterior wall (such as Box ablation) may improve the success rate of AF ablation (21). (2) Voltage mapping strategies are significantly different among different studies. Mapping tools, catheter direction, and especially the contact between catheter and tissue will affect the mapping results, resulting in false-positive results of LVZ. In this study, when the pressure catheter was used for voltage mapping during the operation, the pressure at each measuring point was guaranteed to be greater than 5 g, and the “pseudo-LVZ” was excluded.

In recent years, the relationship between EAT and AF has received much attention. Basic research showed (22, 23) that EAT could lead to fibrosis through fatty infiltration, pro-fibrosis, and inflammatory responses (autocrine and paracrine secretion of pro-inflammatory cytokines). A previous study demonstrated that human EAT could induce atrial fibrosis in rats (12). In clinical studies, it was found that EAT rather than systemic obesity is associated with AF, and EAT is an independent predictor of AF (24–26). In order to exclude the interference of factors such as inflammation, inflammation-related diseases were excluded in this study. Consistent with the above studies it was found that patients with AF had a significantly higher EAT volume and lower attenuation value in this study. Recent studies have shown that left atrial EAT thickness is associated with the occurrence and maintenance of AF (9, 10). Because the thickness of different parts of EAT varies greatly and the repeatability of measurement is poor, this study improves the accuracy by measuring the volume of EAT. This study is the first to investigate the relationship between EAT and LVZ in patients with NVAF. Our study found that LA-EAT volume was closely related to left atrial LVZ and was an independent risk factor, suggesting that left atrial EAT may be involved in atrial fibrosis in patients with NVAF. Interestingly, however, our study shows that the left atrial LVZ is less consistent with the LA-EAT distribution area, especially in the posterior wall, with an overlap rate of only 48.6%. It is suggested that the local infiltration effect of LA-EAT is not the leading cause of atrial fibrosis. We speculate that LA-EAT can secrete various substances through paracrine and autocrine and affect the distant atrial myocardium by regulating ion current and electrical coupling or stimulating fibrosis. This may explain the mechanism of EAT in the occurrence of AF from a new perspective. In addition, it can better explain the study of Nakatani Y et al. (27), which found that even complete isolation of the area where the posterior wall overlaps with LA-EAT could not inhibit the recurrence of atrial fibrillation. Kitagawa et al. (28) showed that the attenuation of EAT was significantly correlated with 18F-deoxyglucose uptake, suggesting that EAT attenuation was related to local inflammation. Evaluation of peri-atrial adipose tissue attenuation using CT imaging is a novel method translated from the assessment of vascular inflammation. Kusayama et al. (22) showed that attenuation values decrease with adipocyte proliferation and increase with adipocyte vascularization and fibrosis. Previous studies have found that lower- adipose attenuation is associated with AF recurrence after catheter ablation, but the specific mechanism is not precise (29, 30). Our study found that LVZ is associated with lower adipose attenuation. Its potential mechanism is related to adipocyte hypertrophy and adipose tissue density. Hypertrophic adipocytes may lead to decreased capillary density, inducing adipose tissue hypoxia, macrophage accumulation, and inflammation (31). In addition, larger adipocytes can also increase metabolic activity and secretion of adipose cytokines (such as activity A and matrix metalloproteinases) (12, 32). In our study, advanced age and left atrial volume index were clinical parameters related to left atrial LVZ. This is consistent with Seewöster et al. (33). Consistent with previous studies (34, 35), the AF group had a higher incidence of gastrointestinal disease, which can lead to arrhythmias through vagal as well as inflammatory stimulation. However, in this study, gastrointestinal disease was not statistically different between the LVZ group and the non-LVZ group, possibly because gastrointestinal disease may just be a trigger for this arrhythmia, it has less effect on the atrial stroma that maintains AF (36). In addition, the sample size of this study was relatively small.

Building a prediction model, we find that LA-EAT attenuation may be a more sensitive marker than LA-EAT volume in predicting LVZ. Previous studies have shown that the presence of LVZ is a strong predictor of AF recurrence after catheter ablation (37, 38). The purpose of individualized AF catheter ablation is to improve the matrix for the existence of LVZ, especially in the area with local fragmentation potential, to improve the ablation effect. LA-EAT provides us with a new idea. Our study shows that the volume and attenuation of LA-EAT are related to the occurrence of left atrial LVZ. Therefore, by measuring EAT under CT before the operation, we can understand the situation of left atrial fibrosis, which has important clinical significance for individualized treatment decision-making of AF before operation.

### Limitations

There are still some limitations in this study. First, this study is a single-center, small sample size study, so a multicenter, large sample size study is needed to explore the factors affecting LA-LVZ in patients with NVAF. Second, because this study is a retrospective study, we did not take blood samples from the coronary sinus to evaluate local inflammatory cytokines. Third, we performed left atrial voltage mapping under CPVI or cardioversion sinus rhythm, which may not be able to determine the LVZ of the pulmonary venous sinus, thus reducing the overall LVZ of the left atrium.

## Conclusion

Patients with AF showed higher volume of EAT and lower attenuation value. The volume and attenuation of LA-EAT are associated with the presence of LVZ, a surrogate for atrial fibrosis, within the myocardium. When predicting the existence of LVZ, LA-EAT attenuation is more valuable than LA-EAT volume.

## Data availability statement

The original contributions presented in this study are included in the article/supplementary material, further inquiries can be directed to the corresponding author.

## Ethics statement

Patient studies were approved by the Ethics Committee of Affiliated Hospital of Xuzhou Medical University. The patients/participants provided their written informed consent to participate in this study.

## Author contributions

YS and LC performed the experimental analysis and wrote the manuscript. WC and CZ performed the radio frequency catheter ablation and mapping of low-voltage zones. CS and CX performed experimental data collection. YS designed this study. All authors contributed to the article and approved the submitted version.

## Conflict of interest

The authors declare that the research was conducted in the absence of any commercial or financial relationships that could be construed as a potential conflict of interest.

## Publisher’s note

All claims expressed in this article are solely those of the authors and do not necessarily represent those of their affiliated organizations, or those of the publisher, the editors and the reviewers. Any product that may be evaluated in this article, or claim that may be made by its manufacturer, is not guaranteed or endorsed by the publisher.

## References

[1] Kirchhof P Benussi S Kotecha D Ahlsson A Atar D Casadei B 2016 ESC guidelines for the management of atrial fibrillation developed in collaboration with eacts. *Rev Espanola Cardiol (English ed).* (2017) 70:50. 10.1016/j.rec.2016.11.033 28038729

[2] Asad Z Abbas M Javed I Korantzopoulos P Stavrakis S. Obesity is associated with incident atrial fibrillation independent of gender: a meta-analysis. *J Cardiovasc Electrophysiol.* (2018) 29:725–32. 10.1111/jce.13458 29443438

[3] Lau DH Nattel S Kalman JM Sanders P. Modifiable risk factors and atrial fibrillation. *Circulation.* (2017) 136:583–96. 10.1161/circulationaha.116.023163 28784826

[4] Oikonomou EK Antoniades C. The role of adipose tissue in cardiovascular health and disease. *Nat Rev Cardiol.* (2019) 16:83–99. 10.1038/s41569-018-0097-6 30287946

[5] Goeller M Achenbach S Cadet S Kwan AC Commandeur F Slomka PJ Pericoronary adipose tissue computed tomography attenuation and high-risk plaque characteristics in acute coronary syndrome compared with stable coronary artery disease. *JAMA Cardiol.* (2018) 3:858–63. 10.1001/jamacardio.2018.1997 30027285 PMC6233643

[6] Iacobellis G. Local and systemic effects of the multifaceted epicardial adipose tissue depot. *Nat Rev Endocrinol.* (2015) 11:363–71. 10.1038/nrendo.2015.58 25850659

[7] Iacobellis G Barbaro G. The double role of epicardial adipose tissue as pro- and anti-inflammatory organ. *Horm Metab Res Horm Stoffwechselforschung Horm Metab.* (2008) 40:442–5. 10.1055/s-2008-1062724 18401833

[8] Arora R Knight BP. Epicardial atrial fat: not quite as idle as it looks. *Heart Rhythm.* (2015) 12:266–7. 10.1016/j.hrthm.2014.10.040 25460177

[9] Nakamori S Nezafat M Ngo LH Manning WJ Nezafat R. Left atrial epicardial fat volume is associated with atrial fibrillation: a prospective cardiovascular magnetic resonance 3D dixon study. *J Am Heart Assoc.* (2018) 7:e008232. 10.1161/jaha.117.008232 29572324 PMC5907571

[10] Yorgun H Canpolat U Aytemir K Hazırolan T şahiner L Kaya EB Association of epicardial and peri-atrial adiposity with the presence and severity of non-valvular atrial fibrillation. *Int J Cardiovasc Imaging.* (2015) 31:649–57. 10.1007/s10554-014-0579-5 25466809

[11] Friedrichs K Klinke A Baldus S. Inflammatory pathways underlying atrial fibrillation. *Trends Mol Med.* (2011) 17:556–63. 10.1016/j.molmed.2011.05.007 21763201

[12] Venteclef N Guglielmi V Balse E Gaborit B Cotillard A Atassi F Human epicardial adipose tissue induces fibrosis of the atrial myocardium through the secretion of adipo-fibrokines. *Eur Heart J.* (2015) 36:795a–805a. 10.1093/eurheartj/eht099 23525094

[13] Sawa Y Matsushita N Sato S Ishida N Saito M Sanbe A Chronic HDAC6 activation induces atrial fibrillation through atrial electrical and structural remodeling in transgenic mice. *Int Heart J.* (2021) 62:616–26. 10.1536/ihj.20-703 34054002

[14] Ma J Chen Q Ma S. Left atrial fibrosis in atrial fibrillation: mechanisms, clinical evaluation and management. *J Cell Mol Med.* (2021) 25:2764–75. 10.1111/jcmm.16350 33576189 PMC7957273

[15] Jadidi AS Lehrmann H Keyl C Sorrel J Markstein V Minners J Ablation of persistent atrial fibrillation targeting low-voltage areas with selective activation characteristics. *Circ Arrhythm Electrophysiol.* (2016) 9:e002962. 10.1161/circep.115.002962 26966286

[16] Caixal G Alarcón F Althoff TF Nuñez-Garcia M Benito EM Borràs R Accuracy of left atrial fibrosis detection with cardiac magnetic resonance: correlation of late gadolinium enhancement with endocardial voltage and conduction velocity. *Europace Eur Pacing Arrhythm Cardiac Electrophysiol J Working Groups Cardiac Pacing Arrhythm Cardiac Cell Electrophysiol Eur Soc Cardiol.* (2021) 23:380–8. 10.1093/europace/euaa313 33227129

[17] Hindricks G Potpara T Dagres N Arbelo E Bax JJ Blomström-Lundqvist C 2020 ESC guidelines for the diagnosis and management of atrial fibrillation developed in collaboration with the European Association for Cardio-Thoracic Surgery (EACTS): the task force for the diagnosis and management of atrial fibrillation of the European Society of Cardiology (ESC) developed with the special contribution of the European Heart Rhythm Association (EHRA) of the ESC. *Eur Heart J.* (2021) 42:373–498. 10.1093/eurheartj/ehaa612 32860505

[18] Nakahara S Hori Y Kobayashi S Sakai Y Taguchi I Takayanagi K Epicardial adipose tissue-based defragmentation approach to persistent atrial fibrillation: its impact on complex fractionated electrograms and ablation outcome. *Heart Rhythm.* (2014) 11:1343–51. 10.1016/j.hrthm.2014.04.040 24793457

[19] Hori Y Nakahara S Tsukada N Nakagawa A Hayashi A Komatsu T The influence of the external structures in atrial fibrillation patients: relationship to focal low voltage areas in the left atrium. *Int J Cardiol.* (2015) 181:225–31. 10.1016/j.ijcard.2014.12.034 25528317

[20] Kiedrowicz RM Wielusinski M Wojtarowicz A Kazmierczak J. Predictors of the voltage derived left atrial fibrosis in patients with long-standing persistent atrial fibrillation. *Cardiol J.* (2020): [Online ahead of print], 10.5603/CJ.a2020.0069 32419127 PMC9273256

[21] Bisignani A Overeinder I Kazawa S Iacopino S Cecchini F Miraglia V Posterior box isolation as an adjunctive ablation strategy with the second-generation cryoballoon for paroxysmal atrial fibrillation: a comparison with standard cryoballoon pulmonary vein isolation. *J Interv Cardiac Electrophysiol Int J Arrhythm Pacing.* (2021) 61:313–9. 10.1007/s10840-020-00812-z 32632544

[22] Kusayama T Furusho H Kashiwagi H Kato T Murai H Usui S Inflammation of left atrial epicardial adipose tissue is associated with paroxysmal atrial fibrillation. *J Cardiol.* (2016) 68:406–11. 10.1016/j.jjcc.2015.11.005 26685730

[23] Haemers P Hamdi H Guedj K Suffee N Farahmand P Popovic N Atrial fibrillation is associated with the fibrotic remodelling of adipose tissue in the subepicardium of human and sheep atria. *Eur Heart J.* (2017) 38:53–61. 10.1093/eurheartj/ehv625 26612579

[24] Thanassoulis G Massaro JM O’Donnell CJ Hoffmann U Levy D Ellinor PT Pericardial fat is associated with prevalent atrial fibrillation: the framingham heart study. *Circ Arrhythm Electrophysiol.* (2010) 3:345–50. 10.1161/circep.109.912055 20558845 PMC2953855

[25] Shin SY Yong HS Lim HE Na JO Choi CU Choi JI Total and interatrial epicardial adipose tissues are independently associated with left atrial remodeling in patients with atrial fibrillation. *J Cardiovasc Electrophysiol.* (2011) 22:647–55. 10.1111/j.1540-8167.2010.01993.x 21235672

[26] Wong CX Abed HS Molaee P Nelson AJ Brooks AG Sharma G Pericardial fat is associated with atrial fibrillation severity and ablation outcome. *J Am Coll Cardiol.* (2011) 57:1745–51. 10.1016/j.jacc.2010.11.045 21511110

[27] Nakatani Y Sakamoto T Yamaguchi Y Tsujino Y Kinugawa K. Epicardial adipose tissue affects the efficacy of left atrial posterior wall isolation for persistent atrial fibrillation. *J Arrhythm.* (2020) 36:652–9. 10.1002/joa3.12359 32782636 PMC7411190

[28] Kitagawa T Nakamoto Y Fujii Y Sasaki K Tatsugami F Awai K Relationship between coronary arterial (18)F-Sodium fluoride uptake and epicardial adipose tissue analyzed using computed tomography. *Eur J Nuclear Med Mol Imaging.* (2020) 47:1746–56. 10.1007/s00259-019-04675-z 31897585

[29] Klein C Brunereau J Lacroix D Ninni S Brigadeau F Klug D Left atrial epicardial adipose tissue radiodensity is associated with electrophysiological properties of atrial myocardium in patients with atrial fibrillation. *Eur Radiol.* (2019) 29:3027–35. 10.1007/s00330-018-5793-4 30402702

[30] Beyer C Tokarska L Stühlinger M Feuchtner G Hintringer F Honold S Structural cardiac remodeling in atrial fibrillation. *JACC Cardiovasc Imaging.* (2021) 14:2199–208. Epub 2021/06/21. 10.1016/j.jcmg.2021.04.027., 34147453

[31] Goeller M Achenbach S Marwan M Doris MK Cadet S Commandeur F Epicardial adipose tissue density and volume are related to subclinical atherosclerosis, inflammation and major adverse cardiac events in asymptomatic subjects. *J Cardiovasc Comput Tomogr.* (2018) 12:67–73. 10.1016/j.jcct.2017.11.007 29233634 PMC5776050

[32] Skurk T Alberti-Huber C Herder C Hauner H. Relationship between adipocyte size and adipokine expression and secretion. *J Clin Endocrinol Metab.* (2007) 92:1023–33. 10.1210/jc.2006-1055 17164304

[33] Seewöster T Kosich F Sommer P Bertagnolli L Hindricks G Kornej J. Prediction of low-voltage areas using modified apple score. *Europace.* (2021) 23:575–80. 10.1093/europace/euaa311 33279992

[34] Gesualdo M Scicchitano P Carbonara S Ricci G Principi M Ierardi E The association between cardiac and gastrointestinal disorders: causal or casual link? *J Cardiovasc Med (Hagerstown, Md).* (2016) 17:330–8. 10.2459/jcm.0000000000000351 26702598

[35] Linz D Hohl M Vollmar J Ukena C Mahfoud F Böhm M. Atrial fibrillation and gastroesophageal reflux disease: the cardiogastric interaction. *Europace Eur Pacing Arrhythm Cardiac Electrophysiol J Working Groups Cardiac Pacing Arrhythm Cardiac Cell Electrophysiol Eur Soc Cardiol.* (2017) 19:16–20. 10.1093/europace/euw092 27247004

[36] Floria M Drug VL. Atrial fibrillation and gastroesophageal reflux disease: from the cardiologist perspective. *World J Gastroenterol.* (2015) 21:3154–6. 10.3748/wjg.v21.i10.3154 25780320 PMC4356942

[37] Verma A Wazni OM Marrouche NF Martin DO Kilicaslan F Minor S Pre-existent left atrial scarring in patients undergoing pulmonary vein antrum isolation: an independent predictor of procedural failure. *J Am Coll Cardiol.* (2005) 45:285–92. 10.1016/j.jacc.2004.10.035 15653029

[38] Yamaguchi T Tsuchiya T Fukui A Kawano Y Otsubo T Takahashi Y Impact of the extent of low-voltage zone on outcomes after voltage-based catheter ablation for persistent atrial fibrillation. *J Cardiol.* (2018) 72:427–33. 10.1016/j.jjcc.2018.04.010 29807864

